# Real-Time Hand Gesture Monitoring Model Based on MediaPipe’s Registerable System

**DOI:** 10.3390/s24196262

**Published:** 2024-09-27

**Authors:** Yuting Meng, Haibo Jiang, Nengquan Duan, Haijun Wen

**Affiliations:** 1 College of Mechanical Engineering, North University of China, Taiyuan 030051, China; duannq@nuc.edu.cn; 2 SIMITECH Co., Xi’an 710086, China; mr.boj@outlook.com

**Keywords:** machine learning, real-time monitoring, gesture recognition, Triple Loss

## Abstract

Hand gesture recognition plays a significant role in human-to-human and human-to-machine interactions. Currently, most hand gesture detection methods rely on fixed hand gesture recognition. However, with the diversity and variability of hand gestures in daily life, this paper proposes a registerable hand gesture recognition approach based on Triple Loss. By learning the differences between different hand gestures, it can cluster them and identify newly added gestures. This paper constructs a registerable gesture dataset (RGDS) for training registerable hand gesture recognition models. Additionally, it proposes a normalization method for transforming hand gesture data and a FingerComb block for combining and extracting hand gesture data to enhance features and accelerate model convergence. It also improves ResNet and introduces FingerNet for registerable single-hand gesture recognition. The proposed model performs well on the RGDS dataset. The system is registerable, allowing users to flexibly register their own hand gestures for personalized gesture recognition.

## 1. Introduction

In daily life, gestures provide an efficient means of communication and play a significant role in interpersonal interactions [1]. With the advancement of technology and the development of human–computer interactions, gesture recognition has gradually gained attention as a natural and intuitive way of interacting [2]. Gesture recognition refers to the analysis and identification of human hand movements and postures by computers, enabling natural interactions between humans and machines. It is a vital research area in the fields of human–computer interaction and computer vision [3], with a wide range of potential applications, such as virtual reality, smart homes, security monitoring, handwriting recognition, medical image analysis, and more. Gesture recognition technologies can be classified into two main categories: sensor-based gesture recognition and image-based gesture recognition [4].

In the early stages, gesture recognition heavily relies on sensor-based methods due to immature hardware and algorithms. Data gloves or electromagnetic waves are commonly used to capture hand movements. For instance, IBM introduced a device called “DataGlove” [5], which monitored hand movements in real-time and transmitted them to computers. While this approach often provided more accurate results, the need to wear sensor devices limited its widespread adoption in everyday applications.

In recent years, with the rapid development of computer vision technology, the use of visuals in the industry is becoming increasingly widespread [6,7,8]. The methods for recognizing target movements and postures through image or video analysis are becoming more mature. Compared to traditional methods, image-based gesture recognition does not require additional wearable devices, making the recognition process more natural and gradually becoming mainstream technology [9]. In 2002, a research team at the Massachusetts Institute of Technology (MIT) proposed a vision-based gesture recognition system that used infrared cameras to capture hand movements and employed computer vision techniques for gesture recognition [10]. Subsequently, products such as the Microsoft Kinect sensor and Leap Motion gesture recognizer have made gesture recognition more accessible to the general public [11]. This approach does not require extra equipment and can easily integrate with computers and mobile devices, making it increasingly popular.

Gesture recognition techniques include hand pose representation, feature extraction, and classification algorithms [12]. Hand pose representation methods are typically based on key points, skeletal data, or depth images. Feature extraction methods can utilize shape, texture, or motion-related features of gestures. Classification algorithms may involve traditional machine learning techniques such as SVM (Support Vector Machine) or Random Forests, as well as deep learning algorithms like CNN (Convolutional Neural Networks) or RNN (Recurrent Neural Network) [13].

Image-based gesture recognition can be further classified into two categories: static gesture recognition and dynamic gesture recognition [14,15]. Dynamic gestures refer to gesture sequences over time and space, where hand shape and position change with time. It emphasizes the process of gesture changes. On the other hand, static gestures refer to the posture of the hand at a specific moment, mainly including hand shape, texture, orientation, and relative spatial positions. As static gestures represent a certain state in the process of dynamic gestures, they form the core of gesture recognition. Thus, this paper focuses on exploring static gesture recognition.

Existing methods for static gesture recognition mainly involve custom modeling through traditional approaches and feature extraction and recognition through deep learning methods [16]. Currently, available deep learning models have enhanced the performance of gesture recognition to a certain extent. However, adding a new gesture requires retraining the existing models, and each new gesture demands a substantial amount of data for support. Current methods treat each gesture as a separate class, similar to distinguishing between apples and cats. However, in reality, each gesture falls under the broader category of “hand”, and they share many common characteristics. Therefore, we propose using neural networks to train a classifier for the “hand” category, learning the differences between various gestures, and when encountering a new gesture, the network can recognize and classify it accordingly. By inputting a diverse set of gestures into the neural network, we enabled the model to discern the commonalities and essence of different gestures and learn the distinctions between various and similar gestures. Ultimately, this approach achieves the recognition of new gestures, completing a register-based gesture recognition system.

## 2. Related Work

There are many existing datasets for sign language recognition tasks. In this section, we first review some existing sign language datasets. Athitsos et al. [17] proposed an American sign language dataset called ASLLVD, consisting of 9800 video samples containing 3300 sign language words. Sincan and Keles proposed a Türkiye sign language dataset called AUTSL, which contained 38,336 video samples, including 226 sign languages performed by 43 different sign language speakers. These videos were recorded in both indoor and outdoor environments. This dataset has color, depth, and skeleton modes. Necati [18] proposed RWTH-PHOENIX-WEATHER-2014T, a German sign language dataset for continuous sign language recognition, which was based on weather forecast videos broadcasted by nine sign language hosts. The training set, validation set, and test set of the dataset contain 7096, 519, and 642 data samples, respectively. The CSL-Daily [19] dataset 3 can be used for continuous sign language recognition and translation tasks. CSL-Daily focuses more on daily life scenarios, including multiple themes such as family life, healthcare, and school life. The training, validation, and testing sets of CSL-Daily contain 18,401, 1077, and 1176 video samples, respectively. These datasets have their own focuses, but they still vary from the different posture situations of the same gesture that this article wants to solve. Therefore, in subsequent work, this article has created a small gesture dataset RGDS to supplement the existing gesture dataset with fewer training samples for different postures of the same gesture.

Generally speaking, constructing gesture recognition and classification models is the main stage of gesture recognition technology. By extracting the spatiotemporal features of gestures and classifying them, the existing technologies can mainly be divided into two categories: traditional methods and deep learning. Most traditional methods use pre-defined templates or sequence similarity matchingschemes. Among them, traditional methods such as He Li et al. [14] used the maximum likelihood criterion Hausdorff distance for recognition [15], and used a multi-resolution search strategy to improve the search speed while also recognizing letter gestures as well. However, the recognition effect is not good for gestures that undergo deformation (rotation and scaling). Zhang Liangguo et al. [20] sed the Hausdorff distance template matching method to compare the contour feature points of gesture areas and successfully completed the recognition of 30 Chinese sign language gestures; Yang Xuewen et al. [21] overcame the shortcomings of existing gesture recognition algorithms in handling gestures such as scaling, translation, and rotation, using a method that combines gesture main direction and class Hausdorff distance. Zhu Jiyu et al. [22] used a gesture recognition method based on structural analysis to obtain the overall and local change information of gestures, increasing the types of recognizable gesture types. However, these traditional methods also have common drawbacks, such as relatively complex calculation processes, high manpower consumption, and unsatisfactory real-time performance.

Deep learning methods have now become the mainstream method for completing gesture recognition work: experimental results from studies [23,24,25,26,27,28,29] have shown that gesture recognition technology based on neural network training has the ability to improve the accuracy of some meaningful gesture recognition work to over 95%. Xin Wenbin et al. [23] used a static gesture real-time recognition method based on the ShuffleNetv2-YOLOv3 model, extracted features from gesture images using ShuffleNetv2, and then classified gestures using the YOLOv3 neural network model, increasing the fps from 41 to 44. By adopting the CBAM attention mechanism module, the model’s accuracy increased from 96.6% to 98.2%. Wu [25] adopted a novel recognition algorithm of a dual channel convolutional neural network (DC-CNN) to simultaneously extract features from gesture images and hand images, effectively improving the generalization ability of CNN, but the improvement in accuracy was not very significant. At this point, people noticed the shortcomings of single visual recognition in reading gestures. Songyao Jiang et al. [27] proposed independent sign language recognition (SAM-SLR-v2), a skeleton-aware multimodal framework with a global synthesis model. This pattern provides annotations for skeleton-based sign language recognition, but the recognition performance was poor in cases of occlusion or insignificant posture. Papadimitriou et al. [28] proposed a deep learning framework 3D-DCNN + ST-MGCN using appearance and skeletal information for automatic sign language recognition without special input, which reduced the relative error rate of Greek language recognition by 53%. However, this was only for the recognition of the entire arm and did not show any improvement compared to single-finger or hand recognition.

It can be found in the literature that current deep learning gesture recognition work has developed quite maturely. However, current image-based gesture recognition is based on recognizing the projected image of the hand on the image screen. However, what we generally consider gestures is based on the mutual combination of finger joints, i.e., the same gesture forms different images on the image screen in different poses. Therefore, image-based gesture recognition has limitations in recognizing the same gesture in different poses. In response to the above issues, this method obtains the three-dimensional coordinates of the finger’s joint points and identifies the features of the joint points, enabling users to recognize the same gesture in different postures. Considering the classification of features in gesture recognition models, we did not adopt the commonly used softmax classifier. We found that Florian Schroff et al. [30] used Triple Loss to construct FaceNet, which directly learned the mapping from facial images to a compact Euclidean space, where distance directly corresponded to the measure of facial similarity. The goal of Triple Loss is to make the embeddings of samples with the same label as close as possible in the embedding space and the embeddings of samples with different labels as far apart as possible. The Softmax loss function is commonly used for multi-class classification tasks. It maps the output of the network to a probability distribution such that the sum of probabilities for each category is one. The objective of the Softmax loss function is to maximize the probability of the correct category while minimizing the probability of the incorrect category. However, the Softmax loss function may be troubled when dealing with large intra-class differences as it only considers the differences between samples of the same category and ignores the differences between different categories. In contrast, the Softmax loss function mainly focuses on the differences between samples of the same category, while the Triple Lossfunction pays more attention to increasing the similarity between samples of the same category and increasing the degree of difference between different categories.

Finally, the MediaPipe we used in our research is a cross-platform machine learning framework open-source by Google [31] designed to assist developers in building machine learning applications based on visual, audio, and sensor inputs. Finger in MediaPipe is an algorithm module used for hand pose estimation [32]. It can locate and track fingers through input hand images and estimate the three-dimensional posture of fingers. The input of the Finger module is a set of hand images, such as hand images captured by a camera or hand images that have already been captured. The Finger module detects and tracks fingers, obtaining the position and posture information of each finger. Based on deep neural networks, it trains on a large amount of hand image data to achieve the accurate detection and tracking of fingers. In addition, the Finger module also utilizes some image processing and computer vision algorithms, such as morphological processing, filters, and Kalman filters, to further improve the accuracy and stability of detection and tracking.

## 3. Research Methodology

### 3.1. Gesture Recognition Process

In the context of image recognition and classification, there are four main tasks: classification, localization, detection, and segmentation. For gesture recognition, we needed to address the tasks of classification, localization, and detection. To achieve this, we used the following steps:

1. Classification and Localization:

Utilize the open-source MediaPipe provided by Google to obtain the position and coordinate points of the hand. This step helps us identify and locate the hand within the image.

2. Data Preprocessing:

Apply geometric transformations to the coordinate points of the hand obtained in the previous step to normalize the data. This normalization step ensures that the hand’s shape and position are consistent and comparable across different images.

3. Feature Embedding:

Use FingerNet to perform embedding on normalized hand data. FingerNet is a deep neural network designed to extract representative features from the hand’s coordinate points.

4. Loss Functions:

Apply the Triple Loss and CrossEntropy Loss functions during the network training phase. The Triple Loss function is responsible for pushing embeddings of samples with the same gesture label closer together in the embedding space and pushing embeddings of samples with different gesture labels farther apart. The CrossEntropy Loss function is utilized for the classification aspect of network training.

**Figure 1:**
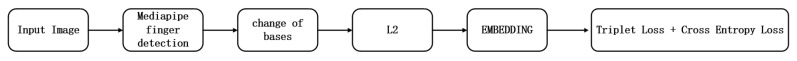
Gesture classification implementation process.

By following these steps (Figure 1), the gesture recognition model can be trained based on the features extracted from hand coordinates, leading to accurate classifying. This approach combines localization and classification to achieve effective gesture recognition.

### 3.2. Data Preparation

For this study, gesture recognition was based on the key points of the fingers. Therefore, if the bending degree of finger joints is the same, it can be considered the same gesture. To address this feature, a registerable gesture dataset (RGDS) was constructed specifically for gesture recognition. The RGDS consists of hand gesture images with a resolution of 2624 × 2457. It contains 32 different gesture categories, each with 50 samples, resulting in a total of 1600 samples.

**Figure 2:**
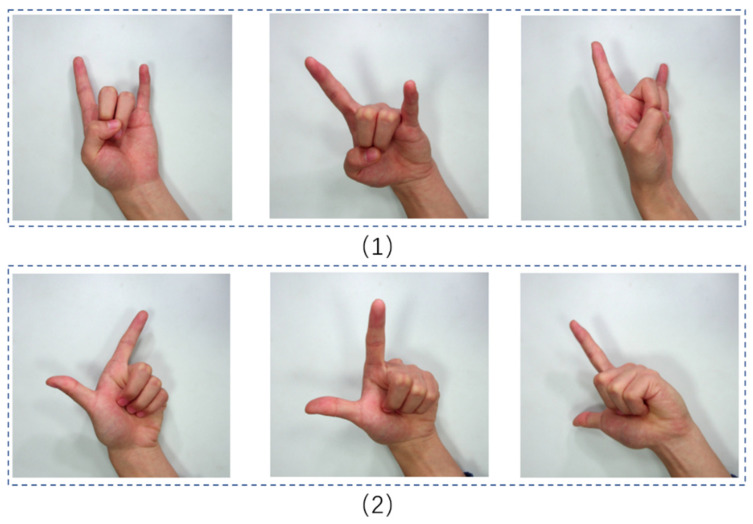
Gesture data. (1) and (2) represent gesture photographs for two different gestures.

As shown in Figure 2, (1) and (2) represent different postures of the same gesture, while (1) and (2) depict different gestures altogether. The RGDS dataset captures these variations in gesture postures and includes images with various finger joint bending degrees, enabling the model to recognize the same gesture in different poses.

Please note that the RGDS dataset has been curated to encompass diverse hand gesture samples, facilitating effective training and the evaluation of the gesture recognition model.

### 3.3. Data Preprocessing

**Figure 3:**
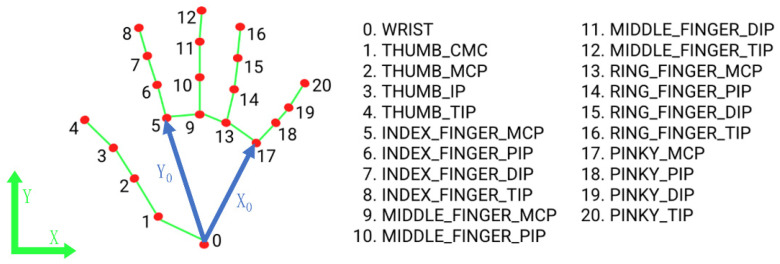
MediaPipe finger landmark. The red dots are the 21 key points selected for the hand, which are connected by a green line to form a complete line of identification of the hand.

After processing the images containing hands through the MediaPipe network, we obtained the three-dimensional coordinates of 21 key points of the fingers, as shown in Figure 3. At this point, the *x* and *y* coordinates of the three-dimensional points are based on the image’s left-bottom corner as the coordinate origin. The z-coordinate is based on the 0th point as the coordinate origin.

To facilitate further image processing, we standardized and rectified the coordinates of the 21 key points of the fingers. Here, we take the 0th point as the coordinate origin; the *x*-axis runs from 0 to 17, and the *y*-axis runs from 0 to 5. Using the right-hand rule, we can obtain a three-dimensional coordinate system based on the 0th point, which will be used for subsequent processing.

Canonical transformations are mathematical transformations commonly used to analyze and simplify the description of physical systems. They involve selecting appropriate coordinate system transformations to convert physical quantities in the original coordinate system, making the problem’s formulation more concise or convenient for solving. The core idea of canonical transformations is to choose a new set of basis vectors to represent vectors in the original coordinate system. These new basis vectors often possess special properties, such as orthogonality or normalization, to simplify the problem’s description or solution.

In this specific case, we are considering two sets of basis vectors: one based on the points 0 to 5 and another based on the points 0 to 17.

(1) Taking the 0th point as the coordinate origin, we performed a translation of the original coordinates. Let *x*_0_, *y*_0_ represent the original coordinates of point 0, and *x_i_*, *y*_i_ represent the original coordinates of point *i*. After the translation, we obtained new coordinates *X*_0_, *Y*_0_.

(1) $$X_{0}=x_{i}-x_{0}$$

(2) $$Y_{0}=y_{i}-y_{0}$$

(2) We calculated the vector $\vec{P_{0}P_{17}}$ and $\vec{P_{0}P_{5}}$

(3) $$\vec{P_{0}P_{17}}=\left(\left(x_{17}-x_{0}\right),\left(y_{17}-y_{0}\right),\left(z_{17}-z_{0}\right)\right)$$

(4) $$\vec{P_{0}P_{5}}=\left(\left(x_{5}-x_{0}\right),\left(y_{5}-y_{0}\right),\left(z_{17}-z_{0}\right)\right)$$

(3) We calculated the normal vector $\vec{P_{z}}$ of the plane formed by vectors $\vec{P_{0}P_{5}}$ and $\vec{P_{0}P_{17}}$

(5) $$\vec{P_{z}}=\vec{P_{0}P_{5}}\times \vec{P_{0}P_{17}}$$

(6) $$\vec{P_{z}}=\left|\begin{array}{ccc} i & j & k\\ x_{5}-x_{0} & y_{5}-y_{0} & z_{5}-z_{0}\\ x_{17}-x_{0} & y_{17}-y_{0} & z_{17}-z_{0} \end{array}\right|$$

(4) Change in Basis

For a point P in the basis of $\vec{Q}=\left(\vec{a},\vec{b},\vec{c}\right)$, its coordinates are given by (*x_r_*, *y_r_*, *z_r_*) using Equation (7). In the basis of $\vec{Q}=\left(\vec{A},\vec{B},\vec{C}\right)$, its coordinates are given by (*x_q_*, *y_q_*, *z_q_*) using Equation (8). The coordinates of $\vec{A}$,$\vec{\;B}$, and $\vec{C}$ in the basis of $\vec{R}\;$ are given, respectively, by (*X_ar_*, *Y_ar_*, *Z_ar_*), (*X_br_*, *Y_br_*, *Z_br_*), and (*X_cr_*, *Y_cr_*, *Z_cr_*) using Equations (9)–(11). Formula (12) can be obtained by Formulas (9)–(11); we obtained Equation (13), which represents the coordinates of point *P* in both bases, showing that (7) = (8). From this, we could derive the coordinate transformation matrix *F* (14) that transforms point *P* from $\vec{R}$ to $\vec{Q}$.

(7) $$P=\left[\begin{array}{c} x_{r}\\ y_{r}\\ z_{r} \end{array}\right]\cdot \vec{R}=\left[\begin{array}{c} x_{r}\\ y_{r}\\ z_{r} \end{array}\right]\cdot \left[\vec{a},\vec{b},\vec{c}\right]=x_{r}\vec{a}+y_{r}\vec{b}+z_{r}\vec{c}$$

(8) $$P=\left[\begin{array}{c} x_{q}\\ y_{q}\\ z_{q} \end{array}\right]\cdot \vec{Q}=\left[\begin{array}{c} x_{q}\\ y_{q}\\ z_{q} \end{array}\right]\cdot \left[\vec{A},\vec{B},\vec{C}\right]=x_{q}\vec{A}+y_{q}\vec{B}+z_{q}\vec{C}$$

(9) $$\vec{A}=\left[\begin{array}{c} X_{ar}\\ Y_{ar}\\ Z_{ar} \end{array}\right]\cdot \vec{P}=X_{ar}\vec{a}+Y_{ar}\vec{c}+Z_{ar}\vec{c}$$

(10) $$\vec{B}=\left[\begin{array}{c} X_{br}\\ Y_{br}\\ Z_{br} \end{array}\right]\cdot \vec{P}=X_{br}\vec{a}+Y_{br}\vec{c}+Z_{br}\vec{c}$$

(11) $$\vec{C}=\left[\begin{array}{c} X_{cr}\\ Y_{cr}\\ Z_{cr} \end{array}\right]\cdot \vec{P}=X_{cr}\vec{a}+Y_{cr}\vec{c}+Z_{cr}\vec{c}$$

(12) $$\vec{Q}=\left[\vec{A},\vec{B},\vec{C}\right]=\left[\begin{array}{ccc} X_{ar} & X_{br} & X_{cr}\\ Y_{ar} & Y_{br} & Y_{cr}\\ Z_{ar} & Z_{br} & Z_{cr} \end{array}\right]\cdot \vec{P}$$

(13) $$\left[\begin{array}{c} x_{q}\\ y_{q}\\ z_{q} \end{array}\right]=\left[\begin{array}{c} x_{r}\\ y_{r}\\ z_{r} \end{array}\right]\cdot \vec{P}\cdot {\vec{Q}}^{-1}=\left[\begin{array}{c} x_{r}\\ y_{r}\\ z_{r} \end{array}\right]\cdot F$$

(14) $$F=\vec{P}\cdot {\vec{Q}}^{-1}$$

### 3.4. Model Construction

**Figure 4:**
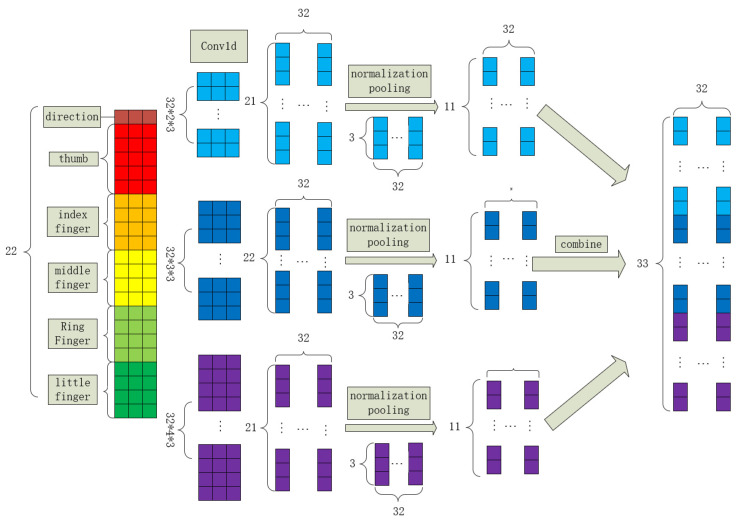
FingerComb block.

After data preprocessing, gesture images were transformed into 1 × 22 × 3-dimensional data. The 22 dimensions comprise the following information: the first dimension represents the hand’s orientation, dimensions 2 to 6 contain the coordinates of each joint of the thumb, dimensions 7 to 10 contain the coordinates of each joint of the index finger, dimensions 11 to 14 contain the coordinates of each joint of the middle finger, dimensions 15 to 18 contain the coordinates of each joint of the ring finger, and dimensions 19 to 22 contain the coordinates of each joint of the little finger. These gesture features are then extracted and combined using the FingerComb block to form 1 × 33 × 32dimensional data. The structure of the FingerComb block is illustrated in Figure 4, where convolution is performed using conv1d.

The main innovation of ResNet-16 lies in the introduction of residual connections, which addresses the issues of gradient vanishing and model degradation during the training of deep networks. This shortcut connection directly adds the input features to the output features, enabling information to flow directly through the network facilitating easier training and optimization.

**Figure 5:**
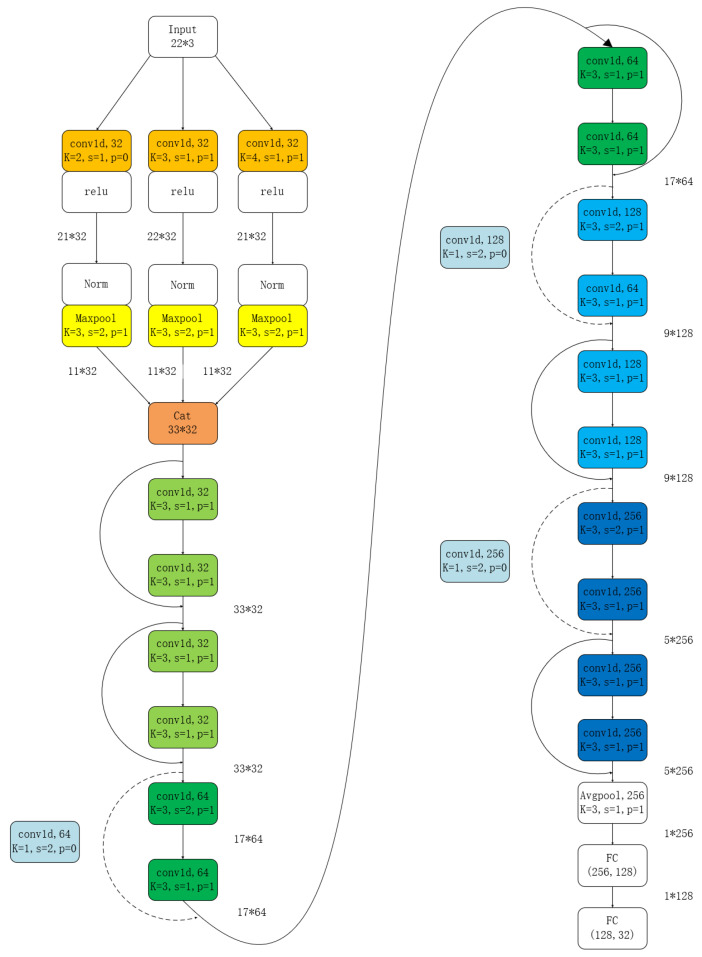
Structure of FingerNet.

In this study, an improved version of ResNet-16, called FingerNet, was achieved by incorporating the FingerComb Block, as shown in Figure 5. The model’s parameters are listed in Table 1. FingerNet takes 1 × 22 × 3-dimensional data as the input and, through convolutional operations, yields a final 1 × 32-dimensional EMBEDDING.

### 3.5. Loss Function

The Triple Loss function enhances the discriminability of gesture features by introducing the concept of triplets. Each triplet consists of an anchor sample (a), a positive sample (p), and a negative sample (n). The objective of the Triple Loss is to minimize the distance between the anchor sample and the positive sample while maximizing the distance between the anchor sample and the negative sample, with the addition of a margin value to ensure increased dissimilarity. By optimizing the Triple Loss function, the network is compelled to learn gesture feature embeddings with better discriminability. Triple Loss performs well in addressing the issue of large intra-class variations, thus improving the accuracy of gesture recognition. However, in practical application, Triple Loss is difficult to train, and the model may struggle to converge. To overcome this, the training process simultaneously employs the CrossEntropy loss function with Triple Loss to improve the model’s convergence speed and gesture recognition performance. This approach balances the optimization of intra-class similarity and inter-class dissimilarity.

Formula (15) represents the Triple Loss, where a denotes the anchor sample, p represents the positive sample, and n represents the negative sample. Formula (16) represents CrossEntropy loss, where m is the number of samples, *W_j_* represents the *j*th weight in the model parameters, *y*_i_ is the true label for each sample, and ${\hat{y}}_{i}$ is the model output. Formula (17) represents the total loss, which is the combination of Triple Loss and CrossEntropy Loss.

(15) $$Lt=\max\left\{d\left(a,p\right)-d\left(a,n\right)+margin,0\right\}$$

(16) $$L\left(w\right)=-\frac{1}{m}\overset{m}{\underset{i=1}{\sum }}y_{i}\left({\hat{y}}_{i}\right)+\frac{1}{m}\overset{m}{\underset{i=1}{\sum }}\left(1-y_{i}\right)\log\left(e^{\frac{1}{m}\Sigma_{j=1}^{m}w_{j}}\right)$$

(17) $$L=Lt+L\left(w\right)$$

## 4. Experimental Procedure and Results

In this section, we analyze the performance of the FingerNet model in the process of gesture recognition through experiments. This model utilizes the RGDS dataset, which we constructed ourselves. The dataset contained a total of 32 classes of gesture data, with 28 classes used as training data and the remaining four classes used for model validation. The hardware and software environment for model training in this study are as shown in Table 2. We built the FingerNet model using the PyTorch framework and trained it using RTX 8000 GPU.

After the completion of the model’s construction, various training parameters needed to be set before training, as shown in Table 3. The model was trained for a total of 500 epochs, and the margin was a parameter for the Triple Loss. Additionally, the “min_tracking_confidence” is the minimum confidence threshold for hand tracking in MediaPipe finger detection, while the “min_detection_confidence” represents the minimum confidence threshold for hand detection. The “max_num_hands” parameter specifies the maximum number of hands to be recognized in the input data.

**Figure 6:**
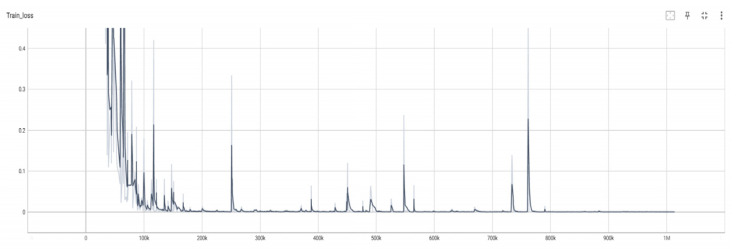
Training process.

After setting up the various training parameters, the model was trained, and Figure 6 shows the change in model training loss with respect to the training steps. Before 200k steps, the model’s loss appears to be unstable but shows an overall convergence trend. After 200k steps, the model’s loss mostly tends to approach zero; although there may be some fluctuations in the process, the loss values are very small.

**Figure 7:**
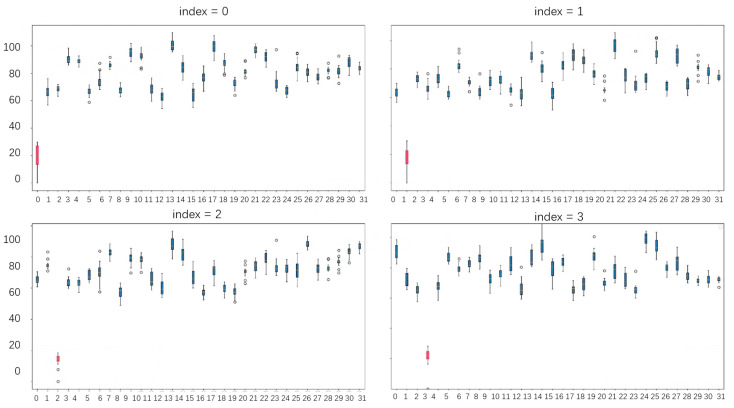
Test results box diagram. The parts marked in red are the gestures in this class that have the smallest L2 distance compared to the other gestures.

After training the model, it was tested using the remaining four classes of hand gestures from the RGDS dataset as registered gestures. The model processed the data and performed cluster analysis on the gestures. The test results are shown in Figure 7, where the *x*-axis represents the 32 classes of hand gestures, and the *y*-axis represents the L2 distance. The figure contains four subplots, each representing the L2 distance between the currently registered gesture and each class in the test data.

For example, in the first subplot, the registered gesture is labeled as the 0th class. From this plot, it can be observed that the L2 distance between the registered 0th class gesture and other 0th class gestures are the smallest, indicating that there is a high intra-class similarity. On the other hand, the distance between the registered gesture and gestures from other classes is larger, indicating a greater inter-class dissimilarity.

Additionally, each class of gestures is well-clustered, showing high intra-class cohesion. Therefore, when setting the threshold to 40, it is possible to completely distinguish between gestures of the same class as the registered gesture and gestures from other classes. This indicates that the FingerNet model is capable of effectively clustering and differentiating between hand gestures based on the learned gesture embeddings.

**Figure 8:**
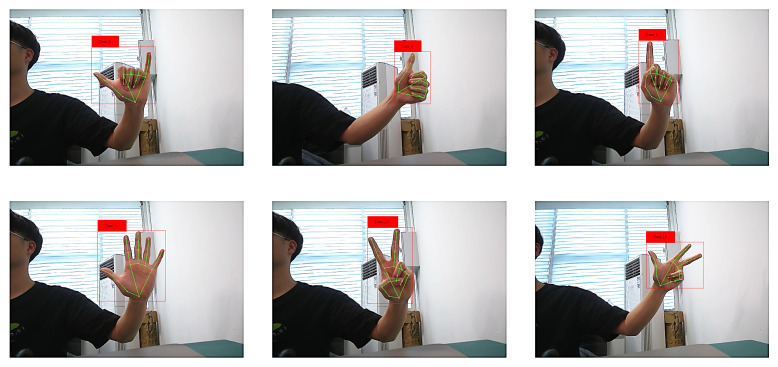
Real-time gesture detection.

After training the model, real-time hand gesture processing and displays were performed using OpenCV to read images from the camera. The images were processed through MediaPipe and then passed to FingerNet for hand gesture recognition. The results are shown in Figure 8.

From this figure, it can be observed that after processing by FingerNet, the model accurately detected the hand positions and hand gesture classes in real-time. Moreover, the model was able to recognize new hand gesture classes through the registration process.

The real-time processing and recognition capabilities of FingerNet demonstrate its effectiveness and practicality in hand gesture recognition applications. The model shows promising performance in accurately identifying hand gestures and can be extended to recognize a wide range of hand movements and poses.

In this paper, in order to further validate the effectiveness of the model method, we also compared some existing models with the model proposed in this chapter on the AUTSL dataset (Table 4), and the results are displayed in accuracy order in the table. Among them, the baseline RGB accuracy comes from the CNN-FPM BiLSTM attention model proposed by Sincan et al. [33], which extracts image features using CNN, fuses multi-scale diffusion convolution through FPM, collects time-domain features using BiLSTM with the attention mechanism, and finally performs time-domain pooling and gesture classifications. The accuracy of LSTM, Transformer, ST-GCN, and SL-GCN methods comes from the OpenHands framework proposed by Selvaraj et al. [34], which mainly uses pre-training and self-supervised learning to improve the accuracy of the model. The remaining models FE + LSTM, MViT-SLR, HWGAT, 3D-DCNN + ST-MGCN, SAM-SLR, and STF + LSTM are from the corresponding papers [28,35,36,37,38,39,40]. These models reflect the best results from the current AUTSL dataset. From the comparison results, it can be seen that the accuracy of the method proposed in this paper reaches 0.953. Although not the best results on this dataset, these models were able to effectively recognize large-scale gestures through multimodal skeleton data.

The sample recording in the AUTSL dataset is clear, the lighting is uniform, the frame rate is high, and there is less dynamic blur, making it suitable for skeleton pose estimation. It can accurately obtain finger positions and recognize gestures using graph convolution methods. However, in some scenarios, the quality of the samples to be recognized is relatively low, which has a significant impact on the estimation of body and hand skeletons, limiting the accuracy of subsequent gesture recognition.

In addition, the video recording time in the ChaLearLAPISOGD gesture dataset is relatively early, the frame rate is relatively low, and there are many samples that lack lighting and blur, which makes skeleton estimation prone to containing many errors. We also conducted comparative tests on the model. Table 5 shows the accuracy comparison results of this method on the dataset.

At the same time, we also conducted further research on another contribution of this article, the registrable dataset RGDS. We tested the existing algorithm models and our model on our dataset and compared them in the smaller-scale dataset. The accuracy is shown in the following Table 6.

## 5. Results and Analysis

In this paper, we constructed a registerable gesture recognition dataset (RGDS) containing 32 different gesture classes, each with 50 images, totaling 1600 gesture images. We proposed a normalization method based on canonical transformations for gesture data to facilitate feature extraction and combination. Additionally, we introduced the FingerComb block for feature extraction and combination, which improved the robustness of gesture features and accelerated model convergence. We made improvements to the ResNet architecture to create the FingerNet model and tested it on the RGDS dataset to evaluate the performance and feasibility of the proposed methods. A large number of experiments show that this method has high accuracy in registered gesture recognition, especially on the dataset we created ourselves with absolute accuracy. The advantage of this is that it provides an effective solution for gesture interaction and operation in practical application scenarios.

However, the current model in this article only focuses on the recognition of single gestures when they can be registered and has weak recognition ability for gestures involving multiple gestures or complex actions (such as gestures made with both hands simultaneously). Moreover, large-scale gesture recognition typically requires the inclusion of a wider range of gesture samples, and the generalization ability and accuracy of improvement of this model for large-scale gesture recognition are not very good. In the future, we plan to further promote and improve this model by achieving the registrable recognition of hand gestures and enhancing the model’s ability to understand complex gestures.

## Figures and Tables

**Table 1:** The architecture layer diagram of FingerNet.

Layer Name	Filter	Output Size
Input		1 × 22 × 3
FingerComb	$\left[\begin{array}{cc} 1\times 2, & 32\\ 1\times 2, & \;32\; \end{array}\begin{array}{c} \;\;s=1,p=0\\ \;\;s=1,p=0 \end{array}\right]\;\left[\begin{array}{cc} 1\times 3, & 32\\ 1\times 3, & \;32\; \end{array}\begin{array}{c} \;s=1,p=1\\ \;s=1,p=1 \end{array}\right]\;\left[\begin{array}{cc} 1\times 4, & 32\\ 1\times 4, & \;32\; \end{array}\begin{array}{c} \;s=1,p=1\\ \;s=1,p=1 \end{array}\right]$	1 × 33 × 32
Conv2_x	$\left[\begin{array}{cc} 1\times 3, & 32\\ 1\times 3, & \;32\; \end{array}\begin{array}{c} \;s=1,p=1\\ \;s=1,p=1 \end{array}\right]\times 2$	1 × 33 × 32
Conv3_x	$\left[\begin{array}{cc} 1\times 3, & 64\\ 1\times 3, & \;64\; \end{array}\begin{array}{c} \;\;s=2,p=1\\ \;\;s=1,p=1 \end{array}\right]$ $\left[\begin{array}{cc} 1\times 3, & 64\\ 1\times 3, & \;64\; \end{array}\begin{array}{c} \;s=1,p=1\\ \;s=1,p=1 \end{array}\right]$	1 × 17 × 64
Conv4_x	$\left[\begin{array}{cc} 1\times 3, & 128\\ 1\times 3, & 128 \end{array}\begin{array}{c} \;s=2,p=1\\ \;s=1,p=1 \end{array}\right]$ $\left[\begin{array}{cc} 1\times 3, & 128\\ 1\times 3, & 128 \end{array}\begin{array}{c} \;s=1,p=1\\ \;s=1,p=1 \end{array}\right]$	1 × 9 × 128
Conv5_x	$\left[\begin{array}{cc} 1\times 3, & 256\\ 1\times 3, & 256 \end{array}\begin{array}{c} \;s=2,p=1\\ \;s=1,p=1 \end{array}\right]$ $\left[\begin{array}{cc} 1\times 3, & 256\\ 1\times 3, & 256 \end{array}\begin{array}{c} \;s=1,p=1\\ \;s=1,p=1 \end{array}\right]$	1 × 5 × 256
Avgpool	256 K = 3, s = 1, p = 1	1 × 1 × 256
FC	(256, 128)	1 × 128
FC	(128, 32)	1 × 32

**Table 2:** Hardware and software platforms.

Type	Type Specification
Operating system	Window 11
CPU	Xeon(R) Gold 5218
GPU	NVIDIA Quadro RTX 8000
Deep learning framework	Pytorch 1.13.0
Language	Python 3.7.15
Memory	256 GB
CUDA	11.7
cudnn	8500

**Table 3:** Model training parameters.

Type	Parameter
epoch	500
BatchSize	256
margin	5
lr	0.2
min_tracking_confidence	0.8
min_detection_confidence	0.62
max_num_hands	1

**Table 4:** Comparison results between our method and existing methods on AUTSL.

Method	Accuracy
Baseline RGB	0.425
Baseline RGB-D	0.632
LSTM	0.774
Transformer	0.810
ST-GCN	0.904
SL-GCN	0.919
FE + LSTM	0.934
MViT-SLR	0.957
HWGAT	0.958
3D-DCNN + ST-MGCN	0.984
SAM-SLR	0.985
STF + LSTM	0.986
FINGER-NET	0.953

**Table 5:** Comparison results between our method and existing methods on ChaLearn IsoGD.

Method	Accuracy
Baseline	0.241
Zhu et al. [41]	0.509
Wang et al. [42]	0.555
Li et al. [43]	0.569
FINGER-NET	0.572

**Table 6:** Comparison results between our method and existing methods on RGDS.

Method	Accuracy
LSTM	0.574
Transformer	0.610
Wang et al. [42]	0.706
Li et al. [43]	0.745
StepNet [44]	0.821
FINGER-NET	0.878

## Data Availability

Data is unavailable due to privacy restrictions.
